# Abnormal Origin of the Pulmonary Artery from the Ascending Aorta in the Neonate

**DOI:** 10.21470/1678-9741-2021-0359

**Published:** 2022

**Authors:** Marina Costa Jonas, Cristiane Nunes Martins, Bayard Gontijo Filho, Clarissa de Paula Freitas Rocha Ramalho, Erika Correa Vrandecic, Fernando Amaral

**Affiliations:** 1 Biocor Institute, Nova Lima, Minas Gerais, Brazil; 2 Department of Cardiology, Faculdade de Medicina de Ribeirão Preto, Universidade de São Paulo, Ribeirão Preto, São Paulo, Brazil

**Keywords:** Aorta, Computed Tomography Angiography, General Surgery, Heart Deffects, Congenital, Infant, Newborn, Information, Pulmonary Artery, Research Report

## Abstract

The anomalous origin of one pulmonary artery from the ascending aorta is a rare congenital heart disease, generally diagnosed based on the clinical information and on echocardiographic and computed tomography angiography findings. Here we report two neonates successfully treated with surgery early in life.

**Table:** 

Abbreviations, Acronyms & Symbols	
**Ao**	**= Aorta**
**AO PA**	**= Anomalous origin of pulmonary arteries from the ascending aorta**
**AP**	**= Anteroposterior**
**BCT**	**= Brachiocephalic trunk**
**BTT**	**= Blalock-Taussig-Thomas**
**CN**	**= Case number**
**CPAP**	**= Continuous positive airway pressure**
**CT**	**= Computed tomography**
**IVC**	**= Inferior vena cava**
**LPA**	**= Left pulmonary artery**
**LV**	**= Left ventricle**
**PA**	**= Main pulmonary artery**
**PDA**	**= Patent ductus arteriosus**
**PT**	**= Pulmonary trunk**
**RA**	**= Right atrium**
**RPA**	**= Right pulmonary artery**
**RV**	**= Right ventricle**
**SVC**	**= Superior vena cava**

## INTRODUCTION

The anomalous origin of pulmonary arteries from the ascending aorta (AOPA) is a rare congenital heart disease (*hemitruncus arteriosus*), usually involving the right branch (right pulmonary artery [RPA])^[1,2]^. Here we report two neonates successfully treated early in life.

**Fig. 1:**
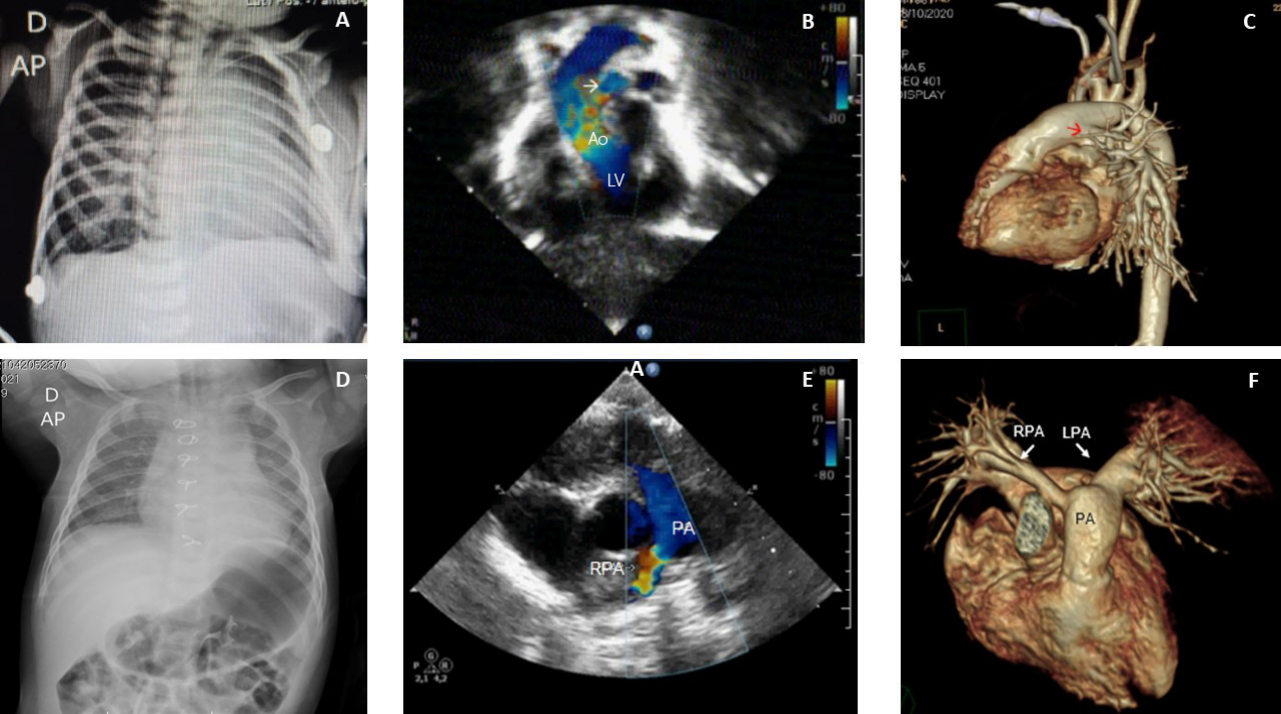
Preoperative (A, B, C) and postoperative (D, E, F) anteroposterior (AP) chest radiography, echocardiogram, and computed tomography angiography in case number 1. Ao=aorta; LPA=left pulmonary artery; LV=left ventricle; PA=main pulmonary artery; RPA=right pulmonary artery.

**Case number CN 1:** A 3.2 kg female neonate with no chromosomal anomalies, absent prenatal issues, Apgar 8/10, and O_2_ saturation 70% required ventilatory assistance in the first hours of life. A systolic murmur was heard, and the chest radiography disclosed an enlarged heart (Figure 1A). Stabilization was possible with O_2_ plus intravenous furosemide and prostaglandin. A transthoracic echocardiogram revealed concordant connections, a patent 3-mm ductus (patent ductus arteriosus [PDA]), and RPA connected to the ascending aorta (Figure 1B, arrow), which was confirmed by computed tomography (CT) angiography (Figure 1C, arrow). On the 5^th^ day of life, the child underwent surgical approach with cardiopulmonary bypass, a 2.5 endotracheal tube, and myocardial protection with Custodiol® infusion. Cerebral protection was achieved by inducing moderate hypothermia (28°C), normal arterial flow, and avoiding air embolism. The PDA was ligated, the normal size RPA (4 mm of diameter) was disconnected from the ascending aorta, and an autologous pericardial patch was used to close its aortic orifice. Ascending aorta retraction was achieved with sutures in the aortic wall, avoiding aorta transection. The RPA was anastomosed to the lateral side of pulmonary artery trunk with an oblique anastomosis in a retroaortic position without a patch. The procedure was completed without transection of the ascending aorta. Ventilation was done in an assist-control mode with progressive regression of parameters until extubation, followed by a 24-hour continuous positive airway pressure (CPAP). Tracheoesophageal fstulation was not noted. Postoperative pulmonary hypertension crises were successfully treated with inhaled nitric oxide, and the patient was discharged 10 days after surgery. At eight months of age, she is asymptomatic on no medication, cardiovascular examination is normal, and neurological status is satisfactory. A recent investigation revealed a normal size heart on the chest radiography with a large stomach air bubble (Figure 1D), a mild stenosis at the anastomotic site in the echocardiogram (Figure 1E, arrow), and the RPA connected to the pulmonary trunk on the CT angiography (Figure 1F).

**Fig. 2:**
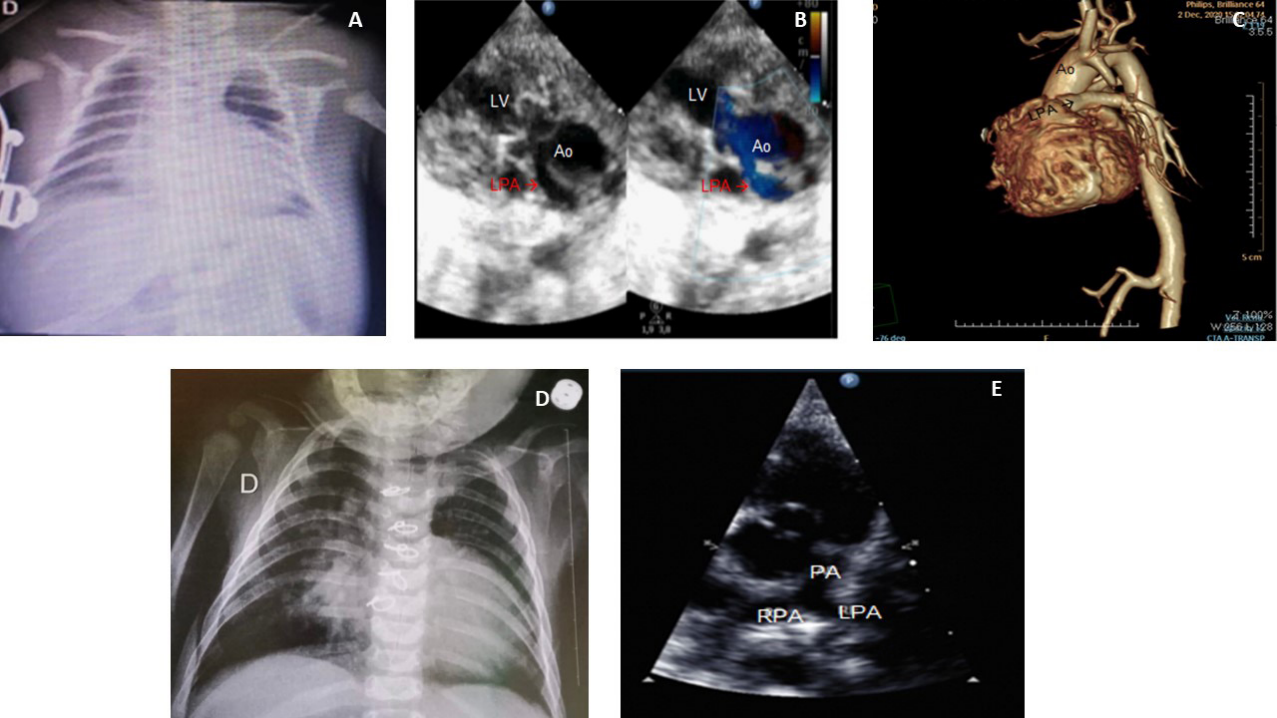
Chest radiography (A), echocardiogram (B), computed tomography angiography (C), and postoperative chest radiography (D) and echocardiogram (E) in case number 2. Ao=aorta; LPA=left pulmonary artery; LV=left ventricle; PA=main pulmonary artery; RPA=right pulmonary artery.

**CN2:** A 2.5 kg male neonate with no chromosomal anomalies and absent prenatal issues was born with pulmonary valve atresia plus a ventricular septal deffect detected on a fetal echocardiogram. Apgar was 7/8 and O_2_ saturation was 78%. A systolic murmur was heard in a eupneic child under intravenous prostaglandin. A chest radiography disclosed an enlarged heart with decreased lung flow (Figure 2A). The echocardiogram showed pulmonary atresia with trunk hipoplasia, a 5-mm ventricular septal deffect, PDA, and the left pulmonary artery (LPA) coming of the ascending aorta (Figure 2B, arrow), which was confirmed by CT angiography (Figure 2C, arrow). Operation occurred on the 6^th^ day of life under cardiopulmonary bypass with aortic and caval cannulation, myocardial protection with Custodiol®, and a 2.5 endotracheal tube. Cerebral protection was achieved by inducing moderate hypothermia (28°C), normal arterial flow, and avoiding air embolism. A previously planned modified right Blalock-Taussig anastomosis using a 4-mm polytetrafuoroethylene tube between the brachiocephalic trunk and the RPA was made with low risk of vocal cord and hemidiaphragm damage. The normal size LPA was occluded at the beginning of bypass, disconnected from the aortic wall, and reimplanted in the pulmonary trunk by means of an oblique sweep anastomosis without a patch. An autologous pericardial patch was used to close the aortic orifice. Ventilation was in an assist-control mode with progressive regression of parameters until extubation, followed by a 24-hour CPAP. Tracheoesophageal fstulation was not noted. The postoperative period was uneventful, discharge occurred 15 days after surgery due to suction disorder (breathing-swallowing incoordination), and birth weight was recovered at 25 days of life. At six months of age, on low aspirin dose and O_2_ saturation of 75%, the neurological status is satisfactory, and a continuous murmur is heard. Recently, the heart was still enlarged on the chest radiography (Figure 2D), and the LPA was connected to the pulmonary trunk on the echocardiogram (Figure 2E).

## QUESTIONS

A) Is it possible to suspect of AOPA based on clinical information?
B) Are the imaging techniques here employed usually enough for diagnosis?
C) Was the surgical treatment ofered in agreement with current practice?

### Discussion of Questions

**Question A:** The clinical picture depends on associated anomalies, but patients often present early with progressive respiratory distress and congestive heart failure, resulting in high mortality rate in the first year^[3]^, as it happens in other deffects. Secondary pulmonary hypertension due to unrestricted aortic flow may lead to cyanosis due to right-to-left shunting through a PDA or a patent foramen ovale^[4]^. When lung flow obstruction is present, respiratory distress will depend on ductus patency, and in CN2, the patient was in a balanced situation. Although a murmur is frequently heard, physical examination is not specific. Unilaterally increased lung flow on the chest radiography may arise diagnostic suspicion.

**Question B:** The transthoracic doppler echocardiogram is useful for diagnosis^[5,6,7,8,9,10]^, and some patients might be operated on based solely on its information^[5,11]^. The subcostal short axis view at the level of the great arteries^[5]^, as well as the suprasternal and short axis longitudinal view, can usually establish the diagnosis. Recently, CT angiography has been employed^[2,4,12]^, and its three-dimensional reconstruction can be used for operative planning, avoiding cardiac catheterization.

**Fig. 3:**
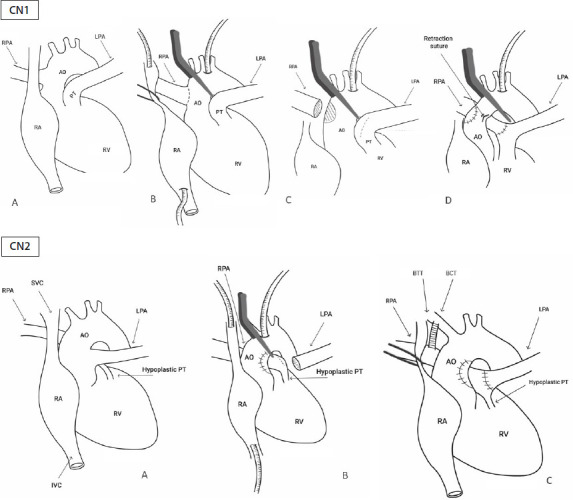
Case number (CN) 1 – A) Right pulmonary artery (RPA) originated from ascending aorta (Ao). B) Aortic and caval cannulation; incision in proximal RPA. C) Disconnection of RPA from aortic wall; closure of aortic orifice with autologous pericardial patch. D) RPA reimplanted in the pulmonary trunk (PT); retraction suture in aortic wall. CN2 – A) Left pulmonary artery (LPA) originated from ascending Ao and hypoplastic PT in pulmonary atresia. B) Aortic and caval cannulation; disconnection of LPA from aortic wall; closure of aortic orifice with autologous pericardial patch. C) Blalock-Taussig-Thomas (BTT) modified shunt; LPA reimplanted in the PT. BCT=brachiocephalic trunk; IVC=inferior vena cava; RA=right atrium; RV=right ventricle; SVC=superior vena cava.

**Question C:** Under cardiopulmonary bypass with moderate hypothermia, both patients had their anomalous pulmonary artery directly implanted in the pulmonary trunk by means of an oblique sweep anastomosis (Figure 3). The procedure is technically demanding, and this operation is recommended to be done early in life. The surgical findings may eventually require an autologous pericardial patch for completing the anastomosis, which can also be accomplished by a synthetic graft or an aortic fap. Associated procedures might also be necessary^[2,4,5,9,11,12,13]^, like in both cases reported, and current surgical mortality is reported to be very low^[2,4,12,14]^.

## BRIEF CONSIDERATIONS OF THE CASES REPORTED

These cases reffect the current recommendations regarding AOPA management in newborns. A high degree of suspicion is necessary to prevent serious consequences, like early pulmonary hypertension and death. Careful echocardiographic examination frequently identifes the anomalous artery, and contemporary investigation by CT angiography is usually enough for surgical planning. Good surgical results are expected but close follow-up is mandatory to detect eventually occurring residual stenosis at the anastomotic site as well as for treatment of associated lesions not yet addressed. As experience increases, very long-term results will appear.
